# Ethical implications of conversational agents in global public health

**DOI:** 10.2471/BLT.19.237636

**Published:** 2020-01-27

**Authors:** David D Luxton

**Affiliations:** a Department of Psychiatry & Behavioral Sciences, University of Washington School of Medicine, 1959 NE Pacific St, Seattle, Washington 98195-6560, United States of America.

The supply of mental health workers in low-income countries is estimated to be as low as 2 per 100 000 population, compared with more than 70 per 100 000 in high-income nations. This shortage in health-care providers results in major gaps in services, contributing to unmet health-care needs and greater disease burden in underserved regions.^1^ Furthermore, in times of humanitarian crisis, such as natural disasters, infectious disease outbreaks or war, the necessity for health-related services arises rapidly and unexpectedly, potentially leaving many thousands of people in need of assistance.

One emerging technology with the potential to help address some shortages and unmet needs not only in mental health, but also for health-care services generally are conversational agents. Conversational agents are software programs that emulate conversation with humans through natural language. Chatbots are the most basic form of a conversational agent and typically communicate with the user through a simple text interface. Embodied conversational agents, or intelligent virtual agents, use computer-simulated virtual characters with the visual appearance of humans or other forms, ranging from simple cartoon-like characters to highly detailed three-dimensional forms.^2^ Speech recognition and natural language processing technologies can allow conversational agents to converse with humans. Affective computing techniques can then provide the conversational agent with the capability to recognize and express emotions, giving them the ability to adapt to the changing states and needs of people while establishing empathetic relationships with them.^2^^,^^3^

Conversational agents are being tested and used to provide and collect health-related information from people, and in some cases, provide treatment and counselling services.^4^^–^^6^ There are, for example, commercially available mobile phone chatbot applications that help people to manage symptoms of depression and anxiety by teaching them self-care and mindfulness techniques. Other use of conversational agents include replacing nurses to provide hospital discharge planning, for collecting relevant health information (such as public health surveys), for training health-care providers by simulating patients, and for providing public health information and education.^3^^,^^5^^–^^9^ Chatbots can also assist health-care professionals by providing them with information about medications, side-effects and other relevant information. Embodied conversational agents are also used in therapeutic computer games and virtual reality environments to provide interactive skills training and treatments for conditions, such as autism spectrum disorder, depression and anxiety disorders.^2^^,^^5^

A benefit of conversational agents is that they can be replicated (scaled-up) easily and affordably to meet demand, and, unlike with human professionals, users can access them via the internet at any time and almost anywhere. They are also potentially more reliable than humans are because they are not affected by fatigue, burnout and cognitive errors.^3^ Conversational agents may also be perceived by users as being free from personal bias. Thus, users may experience less anxiety when discussing private concerns, such as disease exposure risk behaviours, with a conversational agent than they would with another person.^3^^,^^4^^,^^7^ Developers can design conversational agents to have modifiable physical appearance and mannerisms, language and speech dialect, and other characteristics that match them to a user’s cultural background, including race and ethnicity or socioeconomic status. This can help to establish rapport with users, possibly contributing to adherence to treatments and better health outcomes.^3^

## Risk of bias

Despite the potential benefits of the technology, there are ethical challenges to widespread use of conversational agents. The overt appearance and behaviour of conversational agents are susceptible to design biases, such as preference for particular racial or ethnic background in their design. The knowledge base used to train the machine-learning algorithms and the ways that the conversational agent generates new knowledge is also susceptible to bias that is less observable than physical appearance or overt language biases. Algorithmic bias occurs when a software program makes systematic errors that create unfair outcomes, such as privileging one group of users over others. The sources of bias in the data sets used to develop conversational agents may include problems with missing data, misclassification and measurement error and small sample sizes, resulting in underestimation and inaccurate predictions for subgroups of patients. Furthermore, the implicit values of the programmers and organizations who collect, select or use data to train algorithms may also introduce bias.^10^

The application of conversational agents across different countries and different cultures is particularly susceptible to these design biases if the organizations deploying the technology have not adequately considered the demographic characteristics and specific needs of the target end-users. The developers of conversational agents must consider the potential for bias in the design and testing of conversational agents. Including data from the target population and diverse communities within a population who could be affected by disparities in the design, testing and implementation of these technologies is therefore essential. Continual research and evaluation of conversational agents is necessary to help prevent biases and deficiencies in the data used by the machine-learning algorithms that could contribute to socioeconomic disparities in service provision.

## Risk of harm

Since conversational agents function with some level of autonomy, there is a potential risk of harm to people if the technology does not adequately address scenarios when the system detects potential or imminent safety risks. For example, a person conversing with a chatbot could reveal that they are experiencing suicidal thoughts and have immediate plans to die. Also, some patients, such as those with significant psychotic symptoms or cognitive deficits, may not be suitable candidates for the use of conversational agents.^3^

To address these concerns, administrators of the technology should have a process for disclosing its limitations and intended use and for screening users for suitability before they are given access to the conversational agent. Also, the technology should be designed with the capability to monitor for risks automatically and then take appropriate action. In some scenarios, the conversational agent system could provide an immediate display of help resources, such as a crisis line or other helpful resources. In other situations, procedures are needed for a human to review the information and, when appropriate, contact the user to intervene or make an appropriate referral for assistance.

At present, most companies that produce conversational agents for health-related uses market them to the public for providing information or training, and not as replacements for health professionals. Therefore, many of the ethical and legal requirements that human health-care professionals should comply with, such as duty-to-warn, are unheeded. It is, therefore, essential that administrators of these systems provide sufficient information to users regarding the scope of use, risks, limits and expectations regarding the use of conversational agents. Furthermore, administrators of conversational agents must identify and assess the adequacy of resources (such as crisis support or general health-care services) that are available in the countries and regions where the technology will be used.

## Privacy

User privacy is another issue that if not appropriately addressed could potentially cause harm to the users of conversational agents. Conversational agents can collect large amounts of private and sensitive information when people interact with them. Similar to the use of telehealth services, the laws and regulations regarding assurance of user privacy can vary significantly across international borders.^11^ Administrators of conversational agents should be aware of what the local requirements are, if any, and inform end-users of the limits to privacy and of the protections in place to secure users’ data.

## Inequitable access

Realizing the potential of conversational agents in health-care services depends on whether these technologies are accessible to the populations that could benefit from them. Lack of investment in technological infrastructure in underserved communities and countries, as well as the cost of it, is a major limitation to the adoption of any advanced technology.^11^ Lack of education and low technology literacy are other issues that could limit people’s access to and use of conversational agents, resulting in greater health-care disparities across populations. For conversational agents to be viable, therefore, administrators of the technology must consider users’ access to technology and familiarity with its use. As with traditional telemedicine (videoconferencing, telephone consultations and remote monitoring) limitations of the technology or end-user preferences may make conversational agents unsuitable to use. In this case administrators should consider alternatives, such as in-person services.

## Addressing ethical challenges

While conversational agents have the potential to help address some health-service needs worldwide, developers and providers of conversational agent services need to consider the safety, dignity and respect of users to ensure ethical use and application of the technology. One way to start to address the current and emerging ethical challenges associated with the use of conversational agents is to revise or develop new professional ethics codes and practical guidelines.^3^^,^^5^^,^^12^ Most current health profession ethics codes and practice guidelines do not address the use of technologies that simulate and replace human professionals. The World Health Organization could establish a cooperative international working group to review existing ethics principles and guidelines and make recommendations towards assuring the ethical use of artificial intelligence-based tools, including conversational agents and associated technologies worldwide. This work would best be accomplished through collaboration among all stakeholders, including persons who are underserved and most affected by health-care disparities. Concerted advocacy regarding the appropriate use and benefits of conversational agents would also help address concerns about the technology and potentially accelerate its adoption.
